# Developments in Point-of-Care Diagnostic Technology for Cancer Detection

**DOI:** 10.3390/diagnostics8020039

**Published:** 2018-06-02

**Authors:** Bryony Hayes, Caroline Murphy, Aoife Crawley, Richard O’Kennedy

**Affiliations:** 1 Translational Health Sciences, Bristol Medical School, Dorothy Hodgkin Building, Whitson Street, Bristol BS1 3NY, UK; bryony.hayes@bristol.ac.uk; 2 School of Biotechnology, Dublin City University, Collins Avenue, Glasnevin, Dublin D09 Y5N0, Ireland; caroline.s.murphy@dcu.ie (C.M.), aoife.crawley2@mail.dcu.ie (A.C.); 3 Hamad Bin Khalifa University, Research Complex, P.O. Box 34110 Doha, Qatar

**Keywords:** cancer, diagnostics, point-of-care, glycosylation, circulating tumor cells, prostate cancer, biomarkers, exosomes, autoantibodies

## Abstract

Cancer is the cause of death for one in seven individuals worldwide. It is widely acknowledged that screening and early diagnosis are of vital importance for improving the likelihood of recovery. However, given the costly, time-consuming, and invasive nature of the many methods currently in use, patients often do not take advantage of the services available to them. Consequently, many researchers are exploring the possibility of developing fast, reliable, and non-invasive diagnostic tools that can be used directly or by local physicians at the point-of-care. Herein, we look at the use of established biomarkers in cancer therapy and investigate emerging biomarkers exhibiting future potential. The incorporation of these biomarkers into point-of-care devices could potentially reduce the strain currently experienced by screening programs in hospitals and healthcare systems. Results derived from point-of-care tests should be accurate, sensitive, and generated rapidly to assist in the selection of the best course of treatment for optimal patient care. Essentially, point-of-care diagnostics should enhance the well-being of patients and lead to a reduction in cancer-related deaths.

## 1. Introduction: Point-of-Care Diagnostic Tools

Currently, most diagnostic disease testing is carried out in centralized or hospital-based laboratories by using expensive equipment that requires highly trained personnel to operate. To transition towards point-of-care (POC) diagnostics, the tests need to be simplified and miniaturized, which reduces the overall cost of materials, equipment, and personnel costs. The use of “lab-on-a-chip” and biosensor technologies has facilitated this transition, so that a test that was once laboratory-based is now portable and “fit-for-use” by the patients themselves or by “on-site” medical staff [1,2,3,4]. Pai and colleagues envision that a device may not need to be portable so long as the test result is returned quickly and that moving through the “test-and-treat” cycle should be completed as rapidly as possible for the most desirable outcome for the patient [5]. 

Furthermore, factors that are of great significance include cost-effectiveness, capacity to generate real-time results, “simplicity-of-use”, robustness, and functionality without excessive prior-processing of samples. A device that fills these requirements is a biosensor. A biosensor in its simplest terms uses a biological entity (e.g., antibody, enzyme, nucleic acid, lectin, or receptor) to detect an analyte. A transducer then turns the detection signal into an electrical signal that can be quantifiably measured using an appropriate readout [6]. Therefore, biosensors should have great potential to detect changes in the disease state of an individual. This can be achieved by detecting aberrations in biomolecules associated with a person’s genome [7], proteome [8], glycome [9,10], transcriptome [11], metabolome [12], or microbiome [13,14].

In a clinical setting, in order for a diagnostic device to be “fit-for-purpose”, appropriate disease biomarkers must be identified and methods for their analysis incorporated into the device [15]. At present, the detection of biomarkers is used to complement imaging or histopathology, which provides additional information regarding the prognosis or the best treatment options [16,17,18]. However, as stand-alone entities, biomarkers are not yet able to provide definitive diagnoses. Although many biomarkers have been identified, they often fall short of the specificity and sensitivity requirements for clinical diagnostics [19]. This can be attributed to a variety of factors including the wide intra-tumoral and inter-tumoral heterogeneity exhibited from patient to patient [19]. In addition, many “cancer biomarkers” are also elevated in cases of benign disease or may simply be below the limit of detection during the early stages of cancer [4,20]. In these cases, improving technical diagnostic capabilities with regard to detection and more prudent characterization of biomarkers using detailed analysis of post-translational modifications and extending the range of biomarkers to include lipids, metabolites, RNA, or DNA, cells or exosomes may contribute to the development of better biomarker-based technologies and pave the way for biomarkers to become the new gold-standard in diagnostics [11,12,21].

An important operational consideration for POC devices is sample application. Clinical specimens may include saliva, breast milk, urine, cerebrospinal fluid, stool, seminal plasma, amniotic fluid, or blood (which can be further processed by the addition of an anticoagulant and centrifuged forming plasma or by allowing the sample to coagulate followed by centrifugation to remove the blood clot forming serum) [22,23] (see Table 1). The biomarker is present in what is known as the sample matrix, which can greatly impact the results of a POC test. Blood or saliva samples are simple and easy to obtain and may be the preferred options for sample acquisition. However, key factors in sample selection include the presence of the biomarker in the sample in detectable amounts as well as whether or not the detection element (e.g., antibody) can access the biomarker (this may be a problem if the biomarker is membrane bound) and the degree of difficulty or invasiveness involved in obtaining the sample.

Having a greater understanding of the biochemical pathways that are altered during the progression of cancer is leading to the identification of multiple biomarkers. Multiplexing of these biomarkers into a panel gives a greater (diagnostic and prognostic) understanding of the cancer stage and whether or not it will respond to drug treatments [36]. Finally, the determination of the autoantibody signature of a particular cancer using POC diagnostics is currently being recognized as a valuable approach for detecting cancer progression [33]. Some of the previously mentioned biomarkers that are perceived to be important in POC diagnostics will subsequently be critically assessed in greater detail in this review.

## 2. Cancer and the Glycome

Although there are a wide variety of biomarkers available [37,38,39], detection of cancer often requires the use of several biomarkers in tandem to provide a molecular signature sufficiently robust enough to accurately indicate the presence of cancer [33,40,41]. More than 50% of all human proteins are glycosylated [42] and glycosylation is one of the most complex and common post-translational modifications. Given its prevalence in biological systems, it is understandable that changes in glycosylation may cause very undesirable effects in a range of biological systems.

Altered glycosylation is a feature that is common for virtually all types of malignant transformations and disease progression in cancer. However, the types of changes and the extent to which they occur are highly variable. One of the most common aberrancies found is an increase in the branching of N-linked glycans (polysaccharides). For example, extensive β-1,6 branching occurs as a result of upregulated *N*-acetylglucosaminyltransferase V (GnT-V) in breast and colon cancer [43,44]. Other common changes to the glycome observed in cancers include increased cell surface expression of *N*-acetylneuraminic acid and fucose, caused by aberrant activities of sialyl and fucosyltransferases [45]. Ovarian [46], prostate [47], and colorectal cancers [48] have been associated with an increase in fucosylated glycoproteins. The discovery of distinct changes such as these have led to the characterization of specific glyco-profiles in relation to different cancers and significant additional research on how they can be effectively exploited in diagnostics.

## 3. POC Diagnostics for Cancer Detection

### 3.1. Lateral Flow Immunoassays

**Figure 1:**
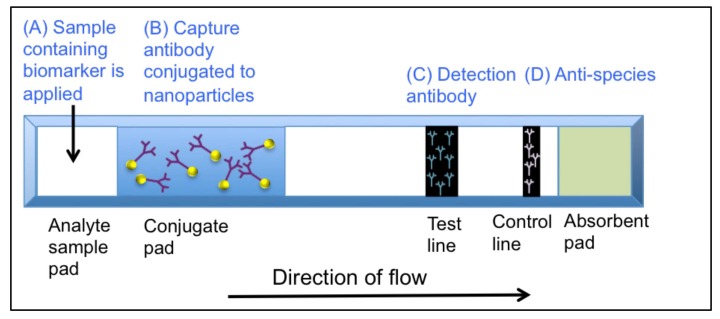
Schematic of a sandwich-format-based lateral flow immunoassay.

Many current POC diagnostics utilize lateral flow immunoassay (LFIA)-based technologies. Lateral flow immunoassays are “dipstick”-like devices that incorporate antibodies to detect the presence of an analyte (e.g., cancer biomarkers). In principle, a lateral flow test is a “simple” device that will provide a qualitative yes/no answer to the presence of a biomarker in a short period of time (usually minutes). It is constructed on a plastic base, which is shown in Figure 1. A sample (usually serum) is applied at position (A). The sample moves along a single axis where it comes in contact with position (B) labeled-capture-antibodies against the biomarker of interest. At position (C), the detection of antibodies at the test line binds to the biomarker and, if present, a visible line will appear at the test line zone. The sample continues to migrate over the control line, which contains anti-species antibodies to detect the capture antibodies and a line appearing at this position indicates that the test worked correctly.

LFIA-based technologies have been successfully used in POC diagnostics and some commercially available examples are shown in Table 2. CTK Biotech’s “Onsite” range of tests include a semi-quantitative rapid test for the prostate cancer-associated marker, prostate specific antigen (PSA). This is an immunochromatographic test that can detect PSA at 4 ng/mL, which is the generally agreed “cut-off” level for the presence of PSA in blood (below 4 ng/mL, is healthy while, above 4 ng/mL, further tests should be considered). The test also indicates that, if a value of 10 ng/mL PSA is found, a biopsy is recommended [49]. The OncoE6™ from Arbor Vita is a lateral flow cervical cancer test device that detects the presence of E6 onco-proteins from high-risk types of human papilloma virus (HPV) types 16 and 18 [50]. However, the test takes 2.5 h, which is a significant length of time for a POC test. INVBIO’s test for the detection of alphafetoprotein (AFP) is a “dipstick”-style test based on a sandwich immunoassay format that detects AFP in serum. It was independently reported that being able to detect AFP below 200 ng/mL (well within the range of INVBIO’s test) is helpful in the diagnosis of hepatocellular carcinoma [51]. Additional examples of commercially available diagnostic tests are outlined in Table 2.

### 3.2. Circulating Tumor Cells

The presence of a cancerous tumor can be detected when peripheral tumor cells are shed and subsequently leave the immediate area of the tumor. They then move through the blood stream or lymphatic system [53]. Some of these cancerous cells (0.01%) have the potential to eventually end up in different parts of the body and give rise to secondary tumors [3,54]. These cells, known as circulating tumor cells (CTCs), are highly amenable to POC detection due to their presence in blood despite often existing in very low numbers (1 in 10^7^–10^9^ cells/mL) [1]. Therefore, specialized isolation techniques (filtration, centrifugation, or immunomagnetic separation) are required for their detection in a POC setting [3]. CELLSEARCH^®^ is one of the first Food and Drug Administration (FDA) approved tests for the detection of CTCs in cancer patients [55]. It detects CTCs displaying epithelial cell adhesion molecules (EpCAM). Magnetic beads functionalized with antibodies against EpCAM are used to extract CTCs from a blood sample [56]. The test can be used to detect metastatic breast, colorectal, or prostate cancers [55]. Isolation of CTCs signals the presence of a tumor, but it can also shed light on the aggressiveness of the disease and whether it will respond well to drug treatment [57]. In addition, it is important to know the extent to which CTCs are present in blood samples since patients with more CTCs appear to have higher mortality rates [58]. However, incorporation of antibodies against a larger number of CTC-derived biomarkers may be necessary to reach the desired levels of sensitivity and specificity.

A device is currently in development that may replace CELLSEARCH^®^ [59,60]. Issadore and colleagues described a method in 2016 to detect CTCs in breast cancer patients [59,60]. The micro-Hall (μ-Hall) chip is designed to be a cost-effective mobile platform for POC use. It utilizes magnetic particles labelled with monoclonal antibodies that target the human epithelial growth factor receptor-2 (HER2), the epidermal growth factor receptor (EGFR), EpCAM, and mucin-1 [60]. The authors suggest that the use of a panel of biomarkers facilitates identification of a heterogeneous population of CTCs. The μ-Hall chip was directly compared to CELLSEARCH^®^ and it could detect 100% of patients compared to only 25% of patients when using CELLSEARCH^®^ [60].

A second device that has the potential to facilitate POC CTC detection is a centrifugo-magnetophoretic system developed by Kirby et al. [61]. In simple terms, the system uses a centrifugal force on a centrifugal disc (CD)-based platform to separate CTCs that are specifically bound to magnetic microbeads. The authors used a MCF7 breast-cancer cell line to mimic CTCs and successfully extracted cells from spiked whole blood. They demonstrated the miniaturization of the separation process onto a “lab-on-a-disc” (LoaD) platform by using a sedimentation driven system that removed the need for the use of filters, pumps, or capillary flow. The system is of low-complexity and uses low sample volumes (18 μL/sample), which makes it highly amenable for use in POC and in low-resource settings.

In 2018, Professor Shana Kelley from the University of Toronto presented the “liquid biopsy” that can detect the presence of prostate cancer derived CTCs. The group have devised a “single cell mRNA cytometry”-based approach [62]. In this approach, cellular mRNA (e.g., *survivin* gene (cell division promoter and apoptosis resistance gene present in many cancer cells)) is targeted by magnetic iron oxide nanoparticles (MNPs). The system uses a pair of probes (important for achieving sufficient magnetic clustering) that are cell permeable and hybridize to the complementary mRNA. Upon hybridization, microscale magnetic clusters are formed and become trapped within the cells. The clusters render the cells magnetic and suitable for capture on a microfluidic device at approximately 10 cells in 1 mL of blood [62]. The system can directly detect mRNAs in CTCs in an amplification-free setting and this approach could be applied toward the detection of CTCs derived from other types of cancer to directly influence therapy [62].

Capturing CTCs using the techniques described can be applied for both signaling the presence of a cancerous tumor and providing details on the stage and severity of the cancer, which supplies the medical practitioner with information that can be used to decide on how best to proceed with the next stage of treatment.

### 3.3. Prostate Cancer

One widely known biomarker used in cancer diagnostics is prostate specific antigen (PSA), which was historically applied to assist in the early identification of prostate cancer (PCa). PSA is a serine protease kallikrein protein produced by epithelial cells in the prostate. However, it is not a specifically a cancer-related marker, and its use has been called into question as a primary screen for prostate cancer [4]. The level of PSA in serum is often raised (>4 ng/mL) in cancer and it has some utility in creating a response to therapy and in longer-term prognosis. It is detectable with the use of an antibody-based POC platform [63]. Uludag and colleagues have developed a “lab-on-a-chip”-based immunosensor platform for PSA detection that encompasses the characteristics of a miniaturized device including automation and low sample volumes. The system is called “MiSens” [2]. The biochip incorporates anti-PSA antibodies immobilized onto an electrochemical-detection-based microfluidic system that can detect PSA at a limit of 0.2 ng/mL. Subsequently, even lower detection levels of PSA were detected by Zhang et al. by utilizing Fabry–Perot interferometer microchips. By incorporating a mouse anti-human-PSA antibody into a nanostructured array, the authors achieved a detection level of 10 pg/mL PSA in complex biological fluids [64]. Both systems mentioned can detect PSA in complex biological fluids well below the level required for disease monitoring.

PSA is, however, not solely a cancer specific biomarker but is a general marker of prostate disease since it is present at elevated levels during benign prostate hyperplasia (BPH) and prostatitis [4]. Many European countries do not run national screening programs for prostate cancer since it may increase the risk of unnecessary treatment for a slowly progressing cancer [65]. Over-diagnosis and unnecessary treatment (i.e., radiotherapy or surgery) can lead to incontinence or impotence, which detrimentally and unnecessarily affects the patients’ quality of life. The alternative to the PSA-based POC test is the digital rectal exam (DRE), which is an unpleasant experience for patients. Therefore, the introduction of a POC test is highly desirable. New biomarkers are urgently needed to replace or complement the use of PSA. Many different forms of PSA exist including elevated levels of complex PSA (cPSA), which are more common in PCa than in other prostate-associated diseases [4]. Therefore, its estimation could further improve the identification of PCa. Moreover, it is known that free PSA (fPSA; non complexed with other proteins) exists in three isoforms, pro-PSA, benign PSA (BPSA) and inactive PSA (iPSA). Studies have suggested that pro-PSA (predominantly composed of [−2]pPSA) is a more cancer-specific isoform that is found in elevated levels at the periphery of the tumor [20,66,67]. Taking the percentage level of [−2]pPSA, total PSA, and fPSA along with a panel of four kallikrein markers, the prostate health index (PHI = [−2]proPSA/fPSA*√tPSA) was developed. The PHI test is an FDA-approved PCa test available from Beckman Coulter, which gives information on the probability of finding prostate cancer in a biopsy. 

An alternative method of PSA detection involves the use of DNA and RNA aptamers (short 10–100 base single-stranded nucleic acid oligomers that can form complex three-dimensional structures [68]). As mentioned, aberrant glycosylation has been implicated as a hallmark of disease state [69,70]. Previous studies have been able to distinguish between healthy men and those with PCa or benign prostatic hyperplasia (BPH) based on differential glycosylation [35,71], which further motivates the determination of cancer-associated glycoforms of PSA. This may ultimately enable the diagnosis of PCa from a blood or urine sample, which would significantly reduce the need for prostate biopsies. In 2016, Jolly et al. used a novel sandwich-based assay for the detection of PCa [72]. The authors used DNA aptamers for the capture of fPSA and developed an aptamer-based enzyme-linked assay that has the potential to detect PSA at a concentration of 0.5 ng/mL and recognize cancer-associated glycoforms. The ability to detect specific glycoforms at 0.5 ng/mL may enable the diagnosis and detection of relapse at an earlier stage. The authors also found that the aptamer-based assay displayed lower cross-reactivity towards human kallikrein 2 (hK2) (which exhibits 80% sequence homology to PSA) when compared to a classic enzyme-linked immunosorbent assay (ELISA) [72]. As with classic ELISA, the technique is performed in a laboratory and requires chemiluminescence-based imaging software to generate results. However, the technique has the potential to be developed into a handheld portable device in the future. 

### 3.4. Pancreatic Cancer

**Figure 2:**
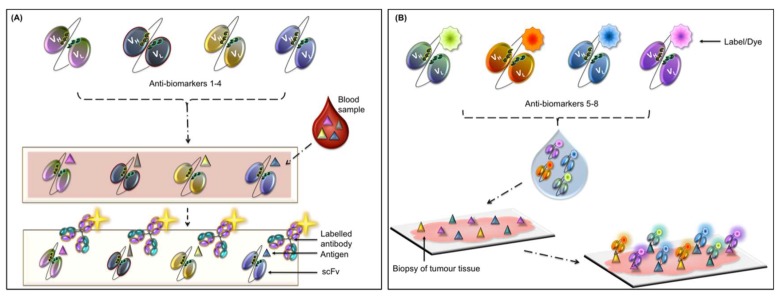
Schematic representation of a multi-marker panel for pancreatic cancer diagnosis. (**A**) This illustration depicts the potential diagnosis of pancreatic cancer using four recombinant antibodies (single chain fragment variable (scFv)) generated against serum antigens found in pancreatic cancer. Each antigen provides information crucial for the treatment and prognosis of the disease. (**B**) Four different scFvs against tissue markers found (or upregulated) in pancreatic cancer are coupled to different dyes or labels. These scFvs will be applied to tumor tissues obtained by a biopsy. Any antigen within the tissue will be detected by the relevant scFv and the levels of each antigen determined. (Adapted from Crawley and O’Kennedy, 2015).

Pancreatic cancer is one of the most aggressive and poorly diagnosed cancers known to date [73]. The aggressive nature and poor prognostic outcomes are a direct result of a combination of factors including chemo-resistance of the tumor, very few symptoms (which are recognizable at stages too late for effective therapy), changing tumor cell behaviors, and its the highly metastatic nature [74]. Pancreatic cancer is associated with mutated genes such as the oncogene (*KRAS*) and the tumor suppressor gene (*TP53*) [36,75]. Additionally, pancreatic cancer is characterized by the progression of precursor lesions with the lesion stage associated with severity/progression of the disease [75]. Crawley and O’Kennedy devised a hypothetical “multi-marker-multi-panel” POC diagnostic test for pancreatic cancer (see Figure 2A and Figure 2B) [36]. They envisioned two panels where panel (A) is a serum-based panel for detecting pancreatic cancer related biomarkers and each biomarker would provide information directly related to the disease [36]. If markers in panel (A) are found to be positive, a biopsy would be performed and the use of proposed panel (B) to provide prognostic information would be initiated. Developing a panel of biomarkers used in combination on a POC device would garner extensive information about the patient’s health in as short a time as possible. The results would directly inform the next stage of therapy. 

## 4. Emerging Biomarkers of Cancer and Their Use in POC Diagnostics

### 4.1. Autoantibody-Based Diagnostics

Aberrant proteins that have been altered (mutated or overexpressed) by cancerous cells known as tumor-associated antigens (TAAs) can evoke an immune system response, which creates cancer-specific autoantibodies. The presence of such antibodies in patients’ sera may be used to signal the presence of cancer at an early stage in the development of the disease [76,77,78]. Autoantibodies are highly stable in serum and can be easily detected and, therefore, make excellent POC targets [76,77,78]. One area where autoantibodies are showing diagnostic potential is in colorectal cancer (CRC). CRC causes approximately 700,000 deaths worldwide every year despite often being curable when diagnosed at an early stage [79]. Screening methods are already in place (fecal occult blood test and colonoscopy) but public uptake is often poor [79]. The reasons for this are many and varied including the uncomfortable nature of the techniques themselves, low sensitivity and low specificity [80], and the relatively high cost of colonoscopy for low resource countries.

A study by O’Reilly et al. investigated the diagnostic potential of a panel of zinc-finger proteins (ZNFs) to detect autoantibodies that may be circulating in the body as a result of colorectal cancer (CRC) [33]. The four ZNF proteins evaluated were ZNF346, ZNF638, ZNF700, and ZNF768, which were over-expressed in approximately 20% of colorectal tumors [81]. The corresponding autoantibodies produced were previously identified using a 37,830-clone recombinant human protein array [82]. For this study, an indirect serum-based ELISA was used. Plates were coated with the ZNF antigens and autoantibodies against each ZNF were detected in the serum of 10% to 20% of CRC patients as compared to 0% to 5.7% of non-cancer participants. The presence of autoantibodies was independent of the disease stage. The sensitivity of the assay for detecting autoantibodies against each individual ZNF facilitated the correct detection of a maximum of 20% of CRC patients. However, examining the presence of autoantibodies against all four ZNFs in a multiplexed approach resulted in an increase in sensitivity of 41.7% compared to only 10% to 20% for each individual ZNF.

These results provide an interesting insight into autoantibodies, which do seem to hold potential for both early screening of patients prior to presentation of symptoms and confirmation of late-stage diagnosis given that autoantibodies are produced irrespective of disease stage. They also highlight the importance of utilizing more than one biomarker for detecting disease.

The identification and incorporation of autoantibodies associated with breast cancer have been discussed by Lacombe et al. in 2014 [83]. Even though mammography is the gold-standard for breast cancer detection, it can lead to over-diagnosis and under-diagnosis in some cases [83]. As previously mentioned, autoantibodies commonly appearing before TAAs can be readily detected and, therefore, incorporating their measurement into POC devices would help in the diagnosis of early-stage breast cancer. Many other papers have discussed the benefits and the use of autoantibodies as potential biomarkers [32] for lung [30], colon [31], liver [84], and stomach cancers [85].

### 4.2. Exosomes

**Figure 3:**
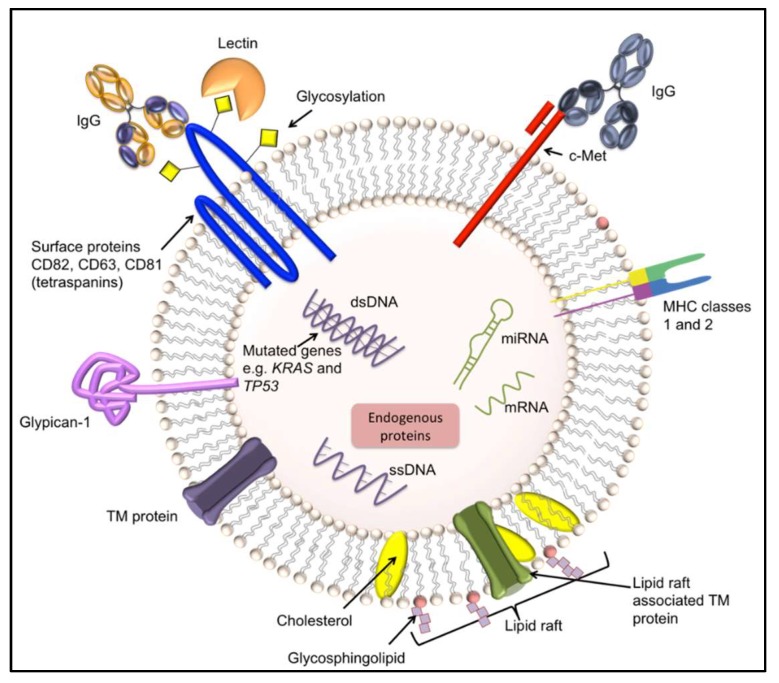
Diagrammatic representation of an exosome. Exosomes are commonly 30–150 nm in diameter. They are composed of a phospholipid bilayer and originate from intraluminal vesicles and are released into the extracellular space. Cancer cells secrete more exosomes than healthy cells and also contain more microRNA (miRNA) [86] (Greening et al., 2015). Exosomes contain mutated genes (e.g., *KRAS* and *TP53*) and lipid rafts (containing high concentrations of cholesterol, glycosphingolipids, and transmembrane proteins (TM)). Glypican-1, which is a cell surface protein, is associated with pancreatic cancer [92] (Melo et al., 2015). Cell surface tetraspanins, e.g., CD82, CD63 and CD81, are commonly used exosome identifiers.

Endosome-derived vesicles or exosomes are small (commonly 30 nm to 150 nm) vesicles excreted by healthy cells and cancer cells and are important for many cellular activities including immunoregulation and cell-cell communication [86,87]. They contain ribonucleic acid (RNA) (messenger RNA and microRNA), double stranded deoxyribonucleic acid (DNA) [88], functional biomolecules, and display surface proteins (e.g., CD63, CD81, and CD82) (see Figure 3). Exosomes have excellent potential for use in POC devices since they are present in extracellular fluids such as blood, plasma, urine, saliva, amniotic fluid, breast milk, and cerebrospinal fluids [21]. They have been found to play an important physiological role in cancer progression [27,86] since c-Met (present on the surface of exosomes) was found to promote angiogenesis and metastasis during melanoma progression in bone marrow-derived progenitor cells [88,89,90]. Furthermore, double stranded DNA (dsDNA) present in exosomes appears to represent the entire genome and is a reflection of the mutational status of the parental tumor cells [88]. Thakur and colleagues compared non-cancerous fibroblasts to cancer cell-lines (myeloma, lung, pancreatic, breast, and prostate cancers) and found a 20-fold increase in exosomal DNA (exoDNA) associated with each cancer cell-line compared to fibroblasts. The authors surmised that exoDNA would be an excellent biomarker candidate for early detection of cancer and metastasis due to protection of the DNA within the exosome and the possible enrichment and detection by antibodies using exosomal surface markers [88]. For bladder cancer diagnosis, to improve upon the gold-standards of cytology (which suffers from low sensitivity) and cystoscopy (which is an uncomfortable invasive procedure), researchers are developing a microfluidic device to isolate and examine exosomes present in urine [27]. The format includes a size-exclusion-based double-filtration microfluidic device for isolation, enrichment, and quantification of exosomes (30 nm to 200 nm) present. The device captures the exosomes and then analyzes them “on-chip” using an anti-CD63-based-ELISA for exosome confirmation. It was found that bladder cancer patients had more exosomes of significantly larger size than healthy controls. The result was captured using a smart phone and the image was transferred to a laptop for data analysis [27]. 

The identification of cancer-specific biomarkers on the surface of exosomes would be of great value in the detection of pancreatic cancer. Previously, genes *KRAS* (oncogene) and *TP53* (tumor suppressor) were found to be mutated in exosomes associated with pancreatic cancer [91]. However, these genes are also associated with other forms of cancer. Subsequently, Melo and colleagues identified glypican-1 (GPC-1) as a specific marker for pancreatic cancer. They found that GPC-1 was most abundant in pancreatic cancer-associated exosomes compared to other cancer-related exosomes. The authors hypothesized that GPC-1 may be a highly valuable biomarker for incorporation onto a POC test and used as a diagnostic biomarker to facilitate non-invasive cancer detection [92].

### 4.3. Lectin-Based Diagnostics

Lectins are a class of proteins that bind to carbohydrates. They have been identified in animals [93], plants [94], and fungi [95]. In humans, lectins play major roles in the innate immune system such as clearing glycoproteins from circulation [96]. They exhibit anti-microbial activities [93,97] and are important in cellular adhesion [98], malignancy, and metastasis [99]. Lectins can recognize glycans (polysaccharides) with varying levels of specificity. Some lectins including recombinant lectins have very high specificity and are able to distinguish between sugar residues at different positions on a particular carbohydrate and between anomeric configurations (α- or β-) [100]. Many lectins have two or more carbohydrate binding domains [101]. For these reasons, lectins are commonly used probes for glycan profiling and the reversible nature of their binding makes them ideal for techniques that use sequential profiling.

In most developed countries, screening programs for cervical cancer are already in place and, as a result, incidence and mortality has declined over the past few decades [102] despite relatively low screening participation rates [103]. Therefore, it is possible that a less intimate method of screening may lead to increased participation and ultimately a further decline in mortality rates. Previous research into serology-based tests for cervical cancer has been largely unsuccessful [104] because more than 10 types of human papilloma viruses (HPV) have been implicated in cervical cancer development [105] and a multiplex assay incorporating many HPV antigens would almost inevitably lead to decreased specificity. In 2016, Jin et al. used an enzyme-linked lectin assay (ELLA) for detecting cervical intraepithelial neoplasia I (CIN I) and cervical cancer using serum immunoglobulins (Ig) [34]. In this study, serology was combined with glyco-profiling to increase specificity with only a single marker. For this, protein A affinity chromatography was used to purify serum Ig, which was then subjected to ELISA to investigate reactivity and ELLA to assess glycosylation [34]. This study [34] found that fucosylation levels of circulating Igs in samples from patients with CIN I and cervical cancer were lower than those in healthy samples. The levels of galactosylation on serum Igs were lower in cancer patients compared with CIN I and healthy samples. However, the levels of sialylation and mannosylation did not differ significantly between groups. Overall, the authors found that reduced fucosylation was an indicator of cervical cancer and that ROC (receiver operating characteristic) curves of both ELLA and ELISA assays found that ELLAs were superior in discriminating CIN I from healthy cells as well as and cervical cancerous cells from healthy cells. However, ELISAs were better able to differentiate between CIN I and cervical cancer [34].

**Figure 4:**
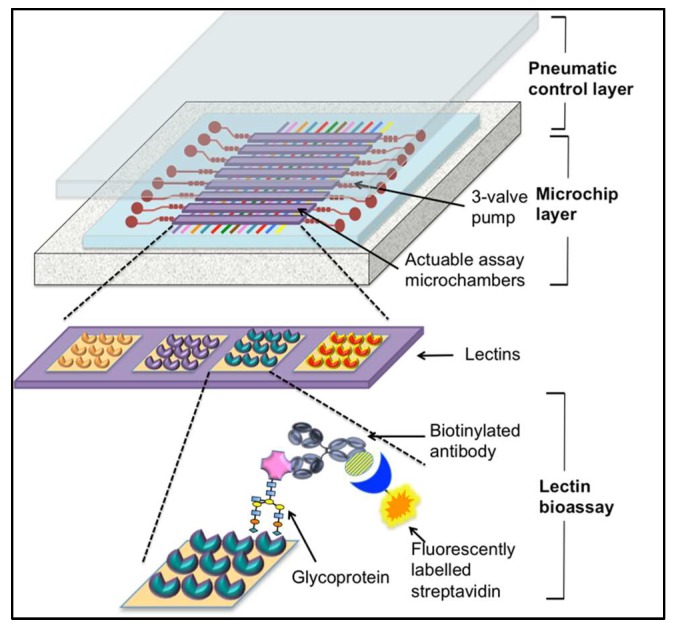
Multi-channel microfluidic lectin-based barcode bioassay. This schematic is a representation of an antibody-lectin sandwich assay on a microfluidic chip. It contains parallel units that have a three-valve pump that promotes fluidic mixing in the assay chamber. Glycoproteins are captured by lectins and detected with biotinylated antibodies against the targeted glycoproteins, which are subsequently identified using fluorescently labelled streptavidin. (Image adapted from Shang et al., 2016).

As an alternative to traditional laboratory-based immunoassay-type methods and lateral flow assays, microfluidics provide some interesting options including smaller sample volumes, quantitative results, and the potential to streamline laboratory protocols for integrating onto a single “chip” [106]. In 2016, Shang et al. developed an automated, microfluidic barcode platform. The system incorporated a sandwich assay using lectins and antibodies to improve sensitivity and provide rapid analysis [107]. The functionality of this assay was demonstrated by the detection of the ovarian cancer biomarker CA125. In this system, microfluidic channels were created in a polydimethylsiloxane (PDMS) membrane integrating eight units in which each consists of three-way micropumps and a lectin micro-barcode assay microchamber (the format for this system can be seen in Figure 4). This membrane is located between a PDMS slab containing a pneumatic control circuit, which promotes fluidic mixing in the assay chambers and enables “stop-flow” fluid transport and a glass slide as the substrate surface patterned with lectin array sensing elements for the capture of glycoproteins. Detection was achieved using biotinylated antibodies targeted by fluorescently labelled streptavidin [107] (see Figure 4).

In the glycomic profiling system developed by Shang et al. a variety of different lectins were used to analyze standard human ovarian CA125 samples from adenocarcinoma tissue (at-CA125), ovarian carcinoma cell lines (cl-CA125), and ascetic fluid from cancer patients (af-CA125). These three samples (plus controls) were run simultaneously to minimize variation (made possible by the multichannel design of this system). The protocol for this involves pumping sample solutions through the microchambers by pneumatic actuation in a “stop-flow” manner. Following lectin capture of glycoproteins and a series of wash and antibody incubation steps, the chip is imaged with fluorescence microscopy using an LED excitation light source. The resulting images were analyzed using ImageJ software to obtain quantitative results [107]. The glyco-profiling results from the three samples tested found that sialylation of proteins shifted from α-2,3-linked to α-2,6-linked following the transition from healthy tissue to cancerous tissue, which was indicated by increased SNA-binding. However, while binding of SNA was observed in ascetic fluid-CA125 and cell-line CA125, almost no reaction was found in adenocarcinoma tissue-CA125. In addition, discrepancies were seen between the three samples including lectins that bind CA125 showed greater affinity for cl-CA125, which suggests increased glycosylation of CA125 in cultured cell lines. Together these results indicate source-sensitive variation in CA125 glycosylation, which should be taken into account. The authors suggest that the improved kinetics observed using this system may be attributed to the confined reaction environment and that the microfluidic design removes limitations such as “mass-transport” [107]. Additionally, despite the considerably smaller sample volume (20 µL vs. 200 µL), the detection of low abundance glycoproteins is still feasible and, with the multichannel system, many samples may be probed simultaneously to provide a glycomic fingerprint and a more comprehensive understanding of specific protein glycosylation in relation to the disease state [107]. However, the total assay time for analysis is up to 3 h, which, while an improvement from ELISAs and other benchtop methodology, is not comparable to rapid POC assays discussed previously.

## 5. Future Directions

Following adjustments and optimizations, many of the techniques described in this review may be adopted for use at POC subsequent to extensive testing through clinical trials to establish sensitivity and specificity data from the general population. Alternatively, diagnostics development may take a completely different approach with techniques such as the use of “smart nanosensors”. Kwon et al. described nanomolecules that can sense their local environment and react to produce a measurable response that is detectable [108]. For example, if the pH in a given area is reduced (due to cellular or tissue damage), the acidity could trigger the release of a payload from a synthetic endosomal-like packaging system [109]. Given that most biomarkers are only present in subsets of different cancer types, it is difficult to imagine an assay that will be universally applicable. Therefore, systems such as these that are able to produce their own indicators to signal disease or provide therapeutic payloads in response to changing cellular or tissue environments may provide the missing link that connects “simple-to-use” POC assays and the high sensitivity and specificity required for a suitable biosensor of cancer. 

The advent of smartphone technology and its presence in every area of the globe has opened up the possibility of utilizing such technologies in POC devices in resource-limited-settings. The smartphone houses many technologies including optical sensor technology as well as the ability to compute complicated algorithms and, therefore, POC tests are not limited to detection by eye but could be used for colorimetric, fluorescent, spectroscopic scattering and microscopy-based approaches [110]. In addition, results can be transmitted instantaneously to initiate treatment if required. However, issues such as the use of different smartphones (e.g., iPhone vs. android), that both differ in their hardware (different camera sensors, phone processors, RAM, and hard drives) and operating systems (some may have options to improve camera image) may contribute to varying results. In addition, changing environmental factors such as lighting could impact the results and, therefore need to be taken into account. Wang and colleagues have achieved the transition from a 96-well plate immunoassay using spectrometer detection to a multichannel smartphone spectrometer (MSS) [111]. The authors address the tremendous need for mobile diagnostic platforms and focused their effort on the development of a miniaturized smartphone-based spectrometer. The team tested the system by assaying the cancer biomarker human interleukin-6 (IL-6) and tackled issues such as the smartphone camera’s limited field of view (FOV), the non-uniform distribution of light across samples, and spectral crosstalk between channels. The authors designed and fabricated a low-cost microprism to increase the FOV, a backlit panel, and a micro-aperture array to achieve uniform light distribution. The result was a multichannel (8 channels) smart-phone-based optical detection platform that could detect a human disease associated biomarker known as IL-6. 

## 6. Discussion

A POC diagnostic device must ideally be cost-effective, rapid, functional without excessive prior-processing of samples, highly sensitive to enable detection of cancer at an early stage, and specific to prevent over-diagnosis, misdiagnosis, or missed-diagnosis. The device must facilitate “self-use” or use by a general practitioner or nurse in a local practice. Results must be returned in a timely manner to initiate treatment as soon as possible, which ultimately leads to the enhancement of the patient’s wellbeing.

Antibody-based systems are a viable option for POC diagnostics, which is demonstrated in their longstanding popularity [6,112]. However, the introduction of lectins and aptamers are making a contribution to POC testing due to their capacity for glycoprofiling [34]. Lectin-based assays such as ELLAs were found to have similar specificity and sensitivity to those seen in antibody-based ELISAs along with their ability to distinguish between glycosylation patterns [34]. Despite the benefits of being small and easy to manipulate, the use of aptamers in diagnostics is still in a developmental stage. However, the increased specificity of aptamers for distinct glycoforms of particular proteins highlights their potential in cancer-associated glycoprofiling and addresses some of the problems encountered with antibodies in identifying cross-reactivity between glycoforms. Therefore, the use of aptamers integrated into assays such as PSA quantification and glycoprofiling has the potential for use in “simple-to-use” POC devices.

The use of biomarker panels to diagnose diseases with greater accuracy is rapidly emerging in addition to the use of autoantibodies due to their physiological relevance, stability, and early appearance in samples. Of equal importance as the identification of novel biomarkers is their incorporation onto miniaturized platforms, which are essentially micro-laboratories fully equipped to receive and process samples as well as produce accurate results. Currently, a concerted global effort is being made to achieve this. 

## Figures and Tables

**Table 1:** Overview of some important sample matrices and associated biomarkers.

Sample Matrix	Biomarkers	Associated Cancer	Ref.
**Saliva**	microRNA panel (miR-9, miR-134, miR-191)	Head and neck squamous cell carcinoma (HNSCC)	[24]
	microRNA panel from whole saliva (miR-10b, miR-144, and miR-451), saliva supernatant (miR-10b, miR-144, miR-21, and miR-451)	Esophageal	[25]
**Urine**	Bence Jones proteins	Light-chain multiple myeloma	[26]
	Exosome size	Bladder	[27]
**Breast milk**	TGF-β	Breast cancer	[28]
**CSF**	CTCs	Metastatic breast cancer giving rise to leptomeningeal metastasis	[29]
**Serum from blood**	PSA	Prostate	[2]
	Autoantibodies	CRC, lung, stomach, breast	[30,31,32]
	ZNF	CRC	[33]
	Igs	CIN I and cervical cancer	[34]
**Seminal plasma**	PSA	Prostate	[35]

Abbreviations: prostate specific antigen (PSA), circulating tumor cells (CTCs), transforming growth factor-beta (TGF-β), colorectal cancer (CRC), zinc-finger proteins (ZNFs), cervical intraepithelial neoplasia I (CIN I), immunoglobulins (Igs), cerebrospinal fluid (CSF).

**Table 2:** Examples of commercially available POC devices for cancer detection. (Adapted from Sharma et al., 2015 [52]).

Associated Cancer	Cancer Biomarker	POC Device	Clinical Capabilities	Test Duration	Sample	Company
Prostate	PSA	PSA Semi-quantitative rapid test	4 ng/mL	15 min	WB, S or P	CTK Biotech
Bladder	Nuclear matrix protein 22 (NMP 22)	Alere NMP22^®^ BLADDERCHEK^®^	99% Sensitivity when combined with cystoscopy	30 min	Urine	Abbott (formerly Alere)
Colorectal	Fecal occult blood	FOB Rapid Test CE	hHB ≥ 50 ng/mL >98% specificity for hHB	5–10 min	Stool	CTK Biotech
Cervical	OncoE6	OncoE6™ Cervical Test	Sensitivity 84.6% Specificity 98.5%	2.5 h	Cervical swab	Arbor Vita
HPV causing head and neck cancer	OncoE6	OncoE6™ Oral Test	Still at testing stage	-	Oral swab	Arbor Vita
Liver	AFP	Medical IVD rapid diagnostic test kits AFP Test kit	Sensitivity 25 ng/mL Specificity 99%	10 min	WB, S or P	INVBIO (Innovation Biotech)
Colorectal, breast, lung,	CEA	CEA Serum Rapid Test	5 ng/mLSensitivity 97%, specificity 100%	10 min	S or P	Cortez Diagnostics Inc.

Abbreviations: Alphafeto protein (AFP), carcinoembryonic antigen (CEA), whole blood (WB), serum (S), plasma (P), human hemoglobin (hHB).
